# *OBSCN* Mutations Associated with Dilated Cardiomyopathy and Haploinsufficiency

**DOI:** 10.1371/journal.pone.0138568

**Published:** 2015-09-25

**Authors:** Steven Marston, Cecile Montgiraud, Alex B. Munster, O’Neal Copeland, Onjee Choi, Cristobal dos Remedios, Andrew E. Messer, Elisabeth Ehler, Ralph Knöll

**Affiliations:** 1 NHLI, Imperial College London, London, United Kingdom; 2 Bosch Institute, The University of Sydney, Sydney, Australia; 3 Randall Division, King’s College London, London, United Kingdom; Centre for Cellular and Molecular Biology, INDIA

## Abstract

**Background:**

Studies of the functional consequences of DCM-causing mutations have been limited to a few cases where patients with known mutations had heart transplants. To increase the number of potential tissue samples for direct investigation we performed whole exon sequencing of explanted heart muscle samples from 30 patients that had a diagnosis of familial dilated cardiomyopathy and screened for potentially disease-causing mutations in 58 HCM or DCM-related genes.

**Results:**

We identified 5 potentially disease-causing *OBSCN* mutations in 4 samples; one sample had two *OBSCN* mutations and one mutation was judged to be not disease-related. Also identified were 6 truncating mutations in *TTN*, 3 mutations in *MYH7*, 2 in *DSP* and one each in *TNNC1*, *TNNI3*, *MYOM1*, *VCL*, *GLA*, *PLB*, *TCAP*, *PKP2* and *LAMA4*. The mean level of obscurin mRNA was significantly greater and more variable in healthy donor samples than the DCM samples but did not correlate with *OBSCN* mutations. A single obscurin protein band was observed in human heart myofibrils with apparent mass 960 ± 60 kDa. The three samples with *OBSCN* mutations had significantly lower levels of obscurin immunoreactive material than DCM samples without *OBSCN* mutations (45±7, 48±3, and 72±6% of control level).Obscurin levels in DCM controls, donor heart and myectomy samples were the same.

**Conclusions:**

*OBSCN* mutations may result in the development of a DCM phenotype via haploinsufficiency. Mutations in the obscurin gene should be considered as a significant causal factor of DCM, alone or in concert with other mutations.

## Introduction

Dilated Cardiomyopathy is a common disease of the myocardium characterised by dilated ventricles and impaired contractility in the absence of any apparent cause, such as coronary artery disease, myocardititis, hypertension, hypertrophic cardiomyopathy or valve disease[1]. Dilated cardiomyopathy frequently leads to heart failure that requires heart transplant.

It is estimated that at least 40% of DCM cases are inherited. Mutations in over 50 genes have been associated with familial DCM, mostly encoding sarcomeric, Z disc and costamere proteins [2]. Truncating mutations in the TTN gene have recently been demonstrated to be the most common cause of FDCM, being identified in around 25% of cases [3, 4]. Studies of the functional consequences of DCM-causing mutations have been limited to a few cases where patients with known mutations had heart transplants [5]. In order to increase the number of potential tissue samples for direct investigation we performed whole exon sequencing of explanted heart muscle samples from 30 patients in the Sydney Heart Bank that had a diagnosis of familial dilated cardiomyopathy. We found potential disease-causing variants in 23 of the patients, mostly in the known DCM-associated genes, including 6 titin truncating mutations. In addition we found 3 mutations in the OBSCN gene that codes for obscurin that suggest this may be a significant cardiomyopathy-associated gene.

Obscurin is a component of the sarcomere that is found bound near the M-line or Z-disk. It has been shown to interact with titin, myomesin and small ankyrin1 and has been proposed to be a structural protein linking the M-line of the sarcomere to the sarcoplasmic reticulum[6–9]. Little is known of it role in the heart: it is reported to be up-regulated in hypertrophic heart [10] but an obscurin knockout mouse exhibited only mild myopathy [11]. Only one mutation in OBSCN has been reported associated with cardiomyopathy; Arimura et al found Arg4344Gln to be related to hypertrophic cardiomyopathy [12]. OBSCN mutations have not previously been reported associated with dilated cardiomyopathy, however, obscurin is expressed in a range of alternatively spliced isoforms in most tissues and OBSCN mutations have been associated with several types of cancer [13, 14].

In this manuscript we have identified OBSCN mutations potentially associated with dilated cardiomyopathy and have investigated the properties of the heart muscles containing OBSCN mutations. We have found that Obscurin expression levels are abnormal when OBSCN is mutated.

## Materials and Methods

We used tissue samples from explanted hearts in the Sydney Heart bank (listed in Supplement A). 30 patients with a diagnosis of familial DCM or idiopathic DCM requiring transplant at a young age were selected for sequencing and further studies. As controls we studied muscle from 6 donor hearts and 3 myectomy samples from patients with HCM (Fig B in S1 File). Donor hearts had no history of cardiac disease and normal ECG and ventricular function and were obtained when no suitable transplant recipient was found. Ethical approval was granted by the Research Integrity, Human Research Ethics Committee, University of Sydney (Protocol No 15401) for collection and distribution of the human heart samples and by the NHS National Research Ethics Service, South West London REC3 (10/H0803/147) for study of the samples. Patients gave written consent with PIS approved by the relevant ethical committee. All samples are anonymised. The investigations conform to the principles of the Declaration of Helsinki. The functional characteristics of some of the donor heart and myectomy samples have been previously reported.[15, 16].

For the genetic investigations we studied a control cohort of patients previously treated with doxorubicin who did not develop heart failure, as previously described [17].

DNA extraction, whole exome sequencing, pyrosequencing, RNA extraction, q-RT-PCR, SDS-PAGE electrophoresis, western blotting and immunofluorescence microscopy were performed by standard methods described in the supplemental methods in S1 File. Obscurin sequences determined were compared with the canonical Obscurin B sequence: RefSeq NP_001092093 and UniProt Q5VST9.

## Results

**Fig 1:**
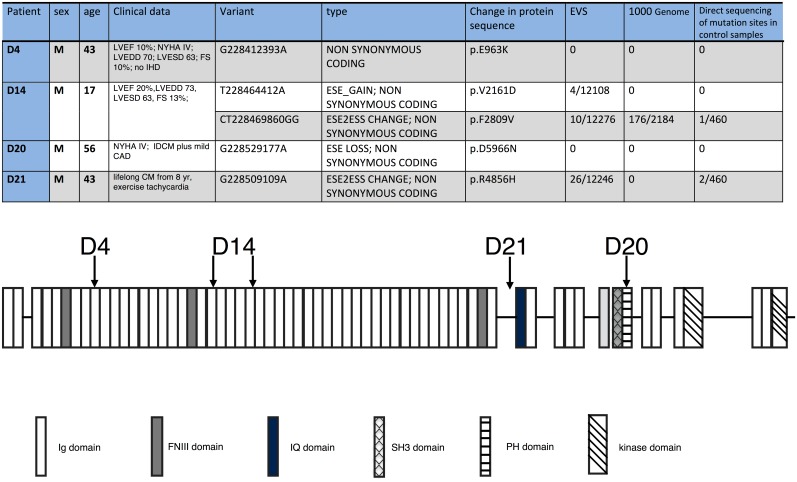
Clinical and genetic data of the 4 heart samples with potentially disease-causing *OBSCN* mutations. The locations of the mutations in the Obscurin B domain diagram are shown, based on the model of Ackerman et al. 2014 (ref 14) (Fig E in S1 File)

We studied muscle samples from 30 explanted hearts of patients with end-stage heart failure and a diagnosis of familial dilated cardiomyopathy. Whole exon sequence data from the patient cardiac muscle samples was screened for potentially disease-causing variants in 58 genes previously implicated in HCM or DCM (Fig C in S1 File). The initial screen identified 5 OBSCN variants in 4 samples (D4, D14, D20 and D21; D14 contained 2 OBSCN variants, Fig 1).

Also identified were 6 truncating mutations in TTN, 3 mutations in MYH7, 2 in DSP and one each in TNNC1, TNNI3, MYOM1, VCL, GLA, PLB, TCAP, PKP2 and LAMA4 (Fig A in S1 File). The sequencing confirms mutations in TNNC1 (D1) and TNNI3 (D2) that have been previously studied [5, 18]. In addition to the OBSCN mutations, D4 also had a DSP mutation and D21 also had a SCN5A mutation. The OBSCN mutations were confirmed by pyrosequencing.

In the “1000 genomes” database one of the mutations in D14 (pF2809V) was found to be present in 14% of genomes and once in our 450 control samples but the other variants were absent. In the Exome Variant Server database, that includes both normal and pathological genomes, both the mutations in D14 were found (pV2161D at 0.03% and pF2809V at 0.08%) and the mutation in D21 was found at 0.2%. All the missense mutations affect fully conserved residues in OBSCN (Fig D in S1 File) except for D21. We therefore excluded D21 as a potential disease-causing mutation.

**Fig 2:**
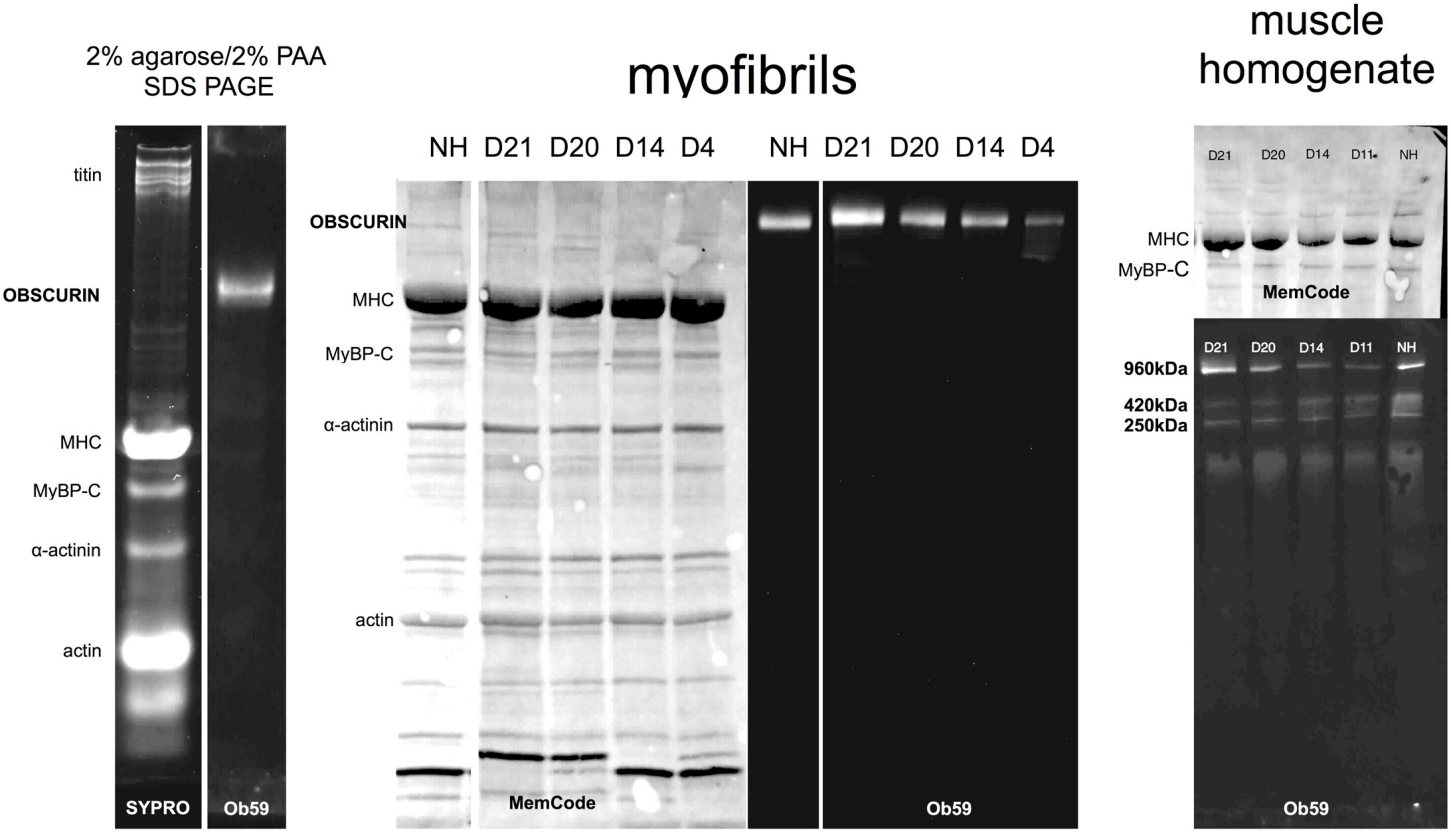
Analysis of obscurin protein expression in tissue samples. Left: Identification of obscurin in cardiac myofibrils by 2% agarose/2% PAA/SDS gel electrophoresis. Left, SYPRO Ruby protein stain, Right Western blot Ob59 antibody. Centre: Obscurin expression in myofibrils. Western blot of 4–18% gradient SDS-PAGE. Left: membrane stained with MemCode protein stain, right: probed with Ob59 antibody. For quantification obscurin was normalised to α-actinin. Right: Identification of obscurin in whole heart muscle homogenates. Top, MemCode protein stain; bottom, Western blot with Ob59 antibody.

**Fig 3:**
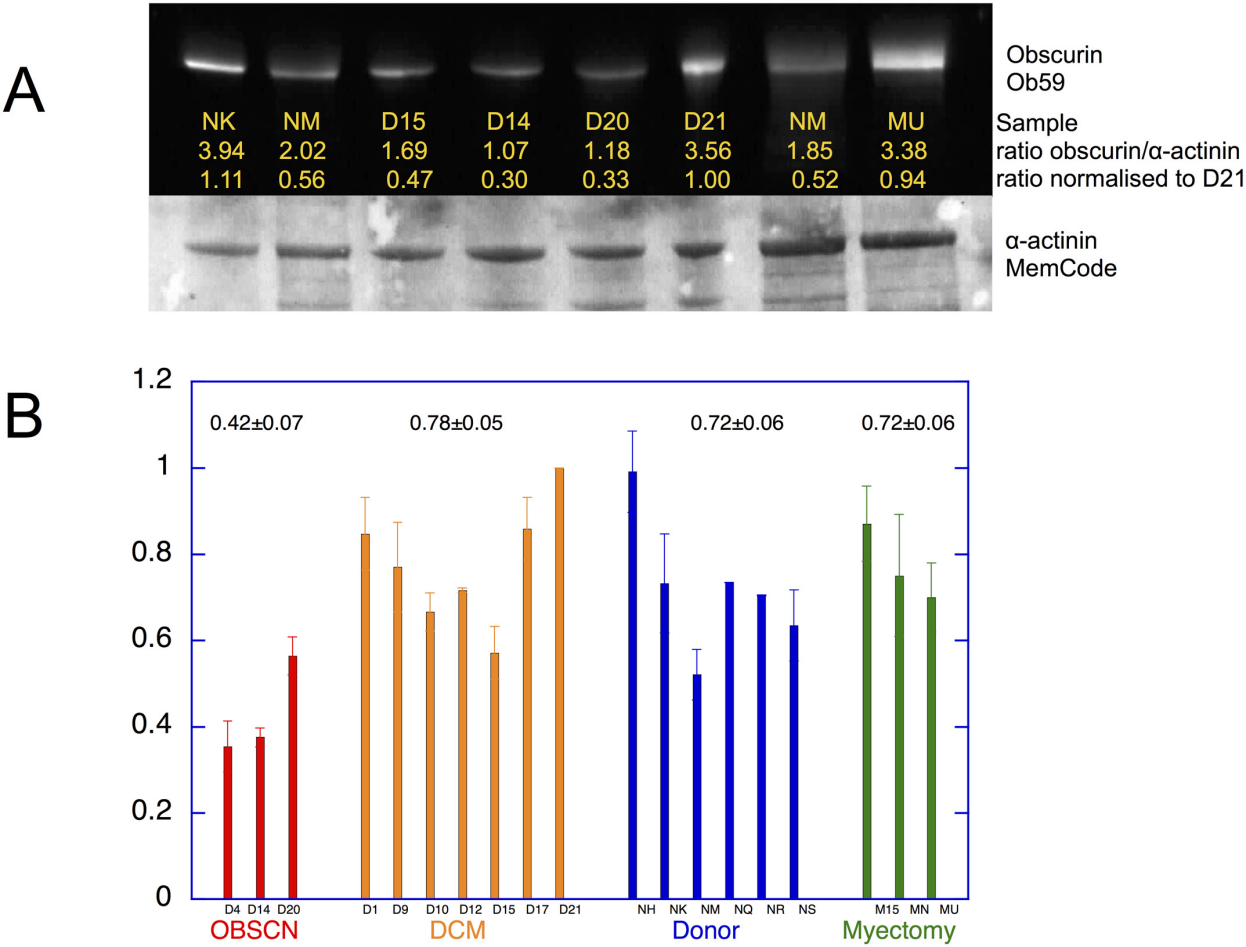
Quantification of Obscurin content of human cardiac myofibrils. A. 4–18% SDS-PAGE of muscle myofibril fraction. Western blot reversibly stained for total protein by MemCode and then probed with Obscurin antibody Ob59. Obscurin band is normalized to the α-actinin band as a loading control and further normalized to D21, present in every slab gel to control for inter-gel variation. B. Relative obscurin protein level normalised to D21. D4, D14, and D20 are compared with 7 DCM samples without obscurin mutations, 6 donor heart samples and 3 myectomy samples (described in S1 File Figs A, B). Data from 22 slab gels similar to that in 2A pooled. The bars represent sem for replicate measurements on the same sample.

We investigated the expression of obscurin in the myofibrillar fraction of human heart muscle samples. The obscurin protein band was barely visible on SYPRO-ruby stained SDS-PAGE gels. Obscurin was positively identified in Western blots using antibodies to domains 13, 58 and 59 ([8], Fig E in S1 File); all three antibodies produced similar results. Obscurin had an apparent mass of around 963±64kDa based on its migration distance on 2% agarose/2% polyacrylamide SDS-PAGE calibrated with titin and Myosin heavy chain. No lower molecular weight peptides were detected in any samples with any antibody. DCM samples with obscurin mutations were indistinguishable from DCM samples without obscurin mutations and donor heart samples on Western blots (Fig 2). The obscurin band in the myofibrillar fraction was quantified on SDS-PAGE relative to α-actinin in the same lane (Fig 3).

Obscurin mutants D4, D14 and D20 exhibited significantly lower levels of obscurin protein compared with the other DCM samples and controls (D4, 45±7%, p = <0.0001, n = 8, D14, 48±3%, p = <0.0001, n = 21 and D20, 72±6%, p = <0.014, n = 14). The level of obscurin in myofibrils was not significantly different between donor, DCM (no OBSCN mutation) and HCM (myectomy) controls.

In heart muscle homogenates multiple isoforms of Obscurin were detected, notably at 960, 420 and 250kDa (Fig 2C). Quantification of the total obscurin (sum of 960,420 and 250kDa bands) revealed substantial obscurin haploinsufficiency in all the Obscurin mutant samples relative to donor heart (Fig G in S1 File). The relative quantities of the three bands was not significantly different between donor and obscurin mutant-containing heart muscle samples, but we noted an interesting regional variation of the proportions of obscurin isoforms. D16 –D30 are atrial samples whilst the D1–D15 and donor and myectomy controls are from left ventricle; we noted that atrial muscle contained significantly more 963kDa band (60.3±5.1%) and less 250kDa band than ventricular tissue (34.3±4.9%, p = 0.00033) (Fig G in S1 File)

We measured obscurin mRNA expression in the cardiomyopathy tissue samples and in controls. The expression of obscurin mRNA is low (ratio = < 0.03) compared to the expression of GAPDH in all samples and variable between the DCM samples and donor heart or HCM heart (myectomy) samples used as control (Fig F in S1 File). When normalised, the mean level of obscurin mRNA was significantly greater and more variable in donor samples than the DCM samples, however the obscurin mRNA content of DCM samples with obscurin mutations was not significantly different from the DCM mutations without obscurin mutations.

**Fig 4:**
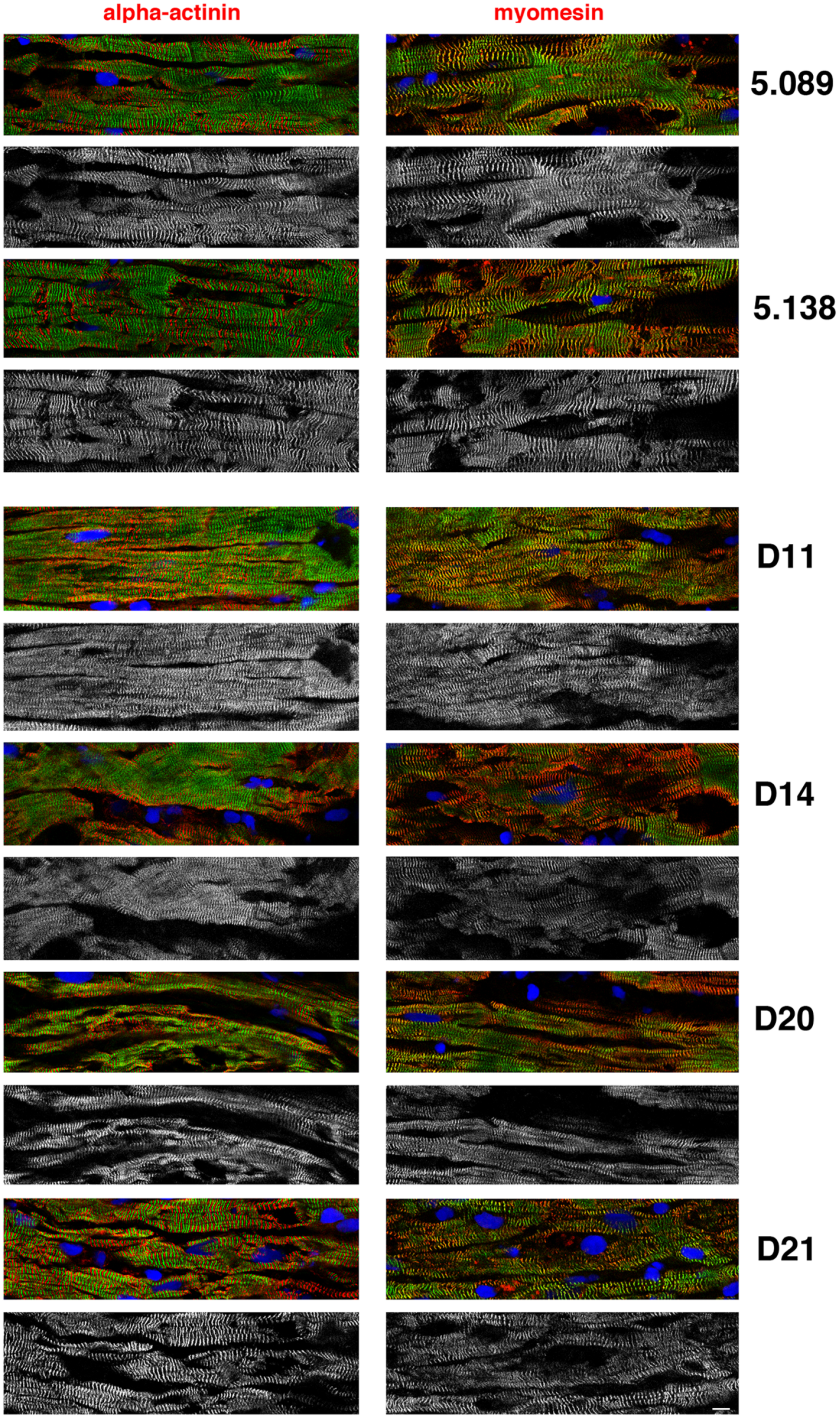
Immunofluorescence images of donor and *OBSCN* mutation- containing human heart tissue sections labelled with Ob59 antibody (green) and anti- α-actinin or anti-myomesin (red). The monochrome panels show the obscurin immunofluorescence on its own.

Finally, we studied the human tissue samples by immunofluorescence microscopy using obscurin, myomesin (M line) and α-actinin (z-disc)-specific antibodies (Fig 4). These clearly showed that obscurin was located at the level of the M-line, coincident with the myomesin label and complementary to the α-actinin (z-disc) label. We could not detect any consistent difference in obscurin quantity or location between samples containing obscurin mutations and donor heart samples.

## Discussion

Whole exome sequencing of explanted heart samples from 30 selected patients with end-stage heart failure, most of which were diagnosed with familial DCM, revealed 26 potentially disease-causing variants in 14 genes, 10 of which have been previously reported ((Fig A in S1 File). This finding of many potentially disease causing mutations in end-stage DCM patients in our small sample correlates with Roberts *et al* finding in a large population that TTN truncation mutations had a >96% probability of pathogenicity when found in end-stage DCM patients [4].

In addition to the known cardiomyopathy-causing genes, we found 4 novel potentially disease-causing variants in the OBSCN gene (Fig 1). This frequency is unexpectedly high since OBSCN mutations have only rarely been linked with any cardiomyopathy. One mutation has been described associated with HCM (in domain 58) and several associated with various forms of cancer[12, 19]. This is thus the first description of an association between OBSCN mutations and dilated cardiomyopathy. We have also investigated obscurin protein levels and have established that the OBSCN mutations are associated with reduced obscurin expression levels, which points to haploinsufficiency as a possible disease causing mechanism.

15% of the potentially disease-causing variants identified in our samples, were in the OBSCN gene; this frequency is similar to that found for TTN truncating mutations that have been proposed as the major gene associated with DCM [4]. Thus, *OBSCN* mutations may also be significant contributors to DCM burden. The reason why *OBSCN* mutations have not been detected before is probably because the large size of the gene has precluded investigation.

One of the variants (D20) was not found in any control population (Fig 1). This mutation is located in the PH domain of obscurin that has been proposed to interact with RhoA. D14 contained two variants in *OBSCN*, one of which (F2809V) was present at 14% in the 1000 genomes database and lower frequencies in the Exon Variants Server (EVS) and our cohort of 460 cancer patients; the other (V2161D) was found at a low frequency in the EVS database only; there is no evidence that the two mutations in D14 have been found together before. It is noted that D4 also contained the *DSP* variant p.R1537C, which was excluded as a disease-causing mutation previously [20]. Mutations in D4 and D14 are in Ig domains with no documented interaction with other proteins. The D21 *OBSCN* mutation is considered not disease-causing since the affected amino-acid is not conserved between species and the mutation is present in control populations; moreover, this sample also contains the *SCN5A* mutation p.S216L, which has been linked to Brugada syndrome[21] and may be a more likely cause of the end-stage heart failure in this patient.

Little is known about obscurin in human heart muscle. When we tested the expression of obscurin in the myofibril fraction of human heart tissue samples we found that Obscurin was present as a single band in all samples with an apparent molecular mass of 960kDa, equivalent to the Obscurin B isoform although smaller, presumably non-muscle, isoforms were observed in the cytoplasm [14]. *OBSCN* variants D4, D14 and D20 were associated with haploinsufficiency in the myofibrillar fraction, with obscurin expression in the range 45–72% of the controls (Fig 3). and this was also apparent in whole tissue (Fig G in S1 File). Parallel measurements of the titin content in the heart samples with titin truncating mutations (Fig A in S1 File) did not show any evidence of titin haploinsufficiency in agreement with [4]. Haploinsufficiency has not previously been reported as a significant factor in DCM but it has been observed in HCM: haploinsufficiency of up to 25% has been shown to be common in *MYBPC3* mutations that cause HCM [22, 23]. Nonsense-mediated mRNA decay and degradation of protein via the ubiquitin-proteasome system have been suggested as major pathways for haploinsufficiency [24], however the mRNA measurements did not provide any evidence for a mutation-related decrease in total obscurin mRNA (Fig F in S1 File). Obscurin is bound to the myofibril through titin and myomesin, and to the sarcoplasmic reticulum through small ankyrin-1[8, 25], consistent with its location at the M-line (Fig 4). Weakening of obscurin binding to the myofibril could cause the observed reduced obscurin content, however this is unlikely to be the cause of the reduced obscurin content of myofibrils since obscurin was also less abundant in whole tissue extract (Fig G in S1 File). Moreover, we did not detect any reduction or relocation of obscurin associated with the mutations by immunofluorescence microscopy (Fig 4).

## Conclusion

We have identified *OBSCN* as a new human cardiomyopathy gene and mutations in this gene should be considered as a significant cause of DCM alone or in concert with another mutation. Disease-related OBSCN mutations cause haploinsufficiency that could account for the development of a DCM phenotype and merit further investigation.

## Supporting Information

S1 File A. Table of muscle samples studied and putative disease-causing mutations found. B. Patient data for the donor heart muscle used as controls in this study. C. WES sequencing protocols: left, list of genes tested for mutations, right filter conditions used to find the potential disease-causing mutations. D. Alignment of Obscurin B isoforms in human, mouse, mole rat and turtle. Only the regions of the sequence with mutations are shown. E. Domain diagram of the two large muscle isoforms of obscurin. Location of mutations and the epitopes of Ob13, Ob58 and Ob59 are shown. F. Relative Obscurin mRNA content of heart muscle samples normalised to GAPDH mRNA. G. Relative obscurin protein expressed in whole homogenates of heart muscle. A) Western blots of whole heart muscle homogenates separated by SDS- PAGE (See Fig 2). Top Memcode protein stain, bottom Ob59 anti-obscurin antibody. B) Calculated relative obscurin levels using the method as shown in Fig 3. Results from individual lanes are shown. C) Relative proportions of the three obscurin bands in atrial and ventricular muscle homogenates.
(PDF)

## Abbreviations

DCM: dilated cardiomyopathy
HCM: hypertrophic cardiomyopathy
IDCM: idiopathic dilated cardiomyopathy
FDCM: familial dilated cardiomyopathy
q-RT-PCR: quantitative reverse transcriptase polymerase chain reaction
SDS-PAGE: sodium dodecyl sulphate polyacrylamide gel electrophoresis
LVEF: left ventricular ejection fraction
LVEDD: left ventricular end-diastolic diameter
LVESD: left ventricular end-systolic diameter
FS: fractional shortening
IHD: ischemic heart disease
CAD: coronary artery disease
EVS: Exon Variants Server
ESE_GAIN: gain of a new exonic splice enhancer element
ESE_LOSS: loss of a exonic splice enhancer element
ESE2ESS_CHANGE: an exonic enhancer element changes into a silencer element
