# Supplementary material for: OBSCN Mutations Associated with Dilated Cardiomyopathy and Haploinsufficiency
Source: PLoS One. 2015 Sep 25;10(9):e0138568. doi: 10.1371/journal.pone.0138568 (PMC4583186; doi:10.1371/journal.pone.0138568)
Supplement: S1 File — A. Table of muscle samples studied and putative disease-causing mutations found. B. Patient data for the donor heart muscle used as controls in this study. C. WES sequencing protocols: left, list of genes tested for mutations, right filter conditions used to find the potential disease-causing mutations. D. Alignment of Obscurin B isoforms in human, mouse, mole rat and turtle. Only the regions of the sequence with mutations are shown. E. Domain diagram of the two large muscle isoforms of obscurin. Location of mutations and the epitopes of Ob13, Ob58 and Ob59 are shown. F. Relative Obscurin mRNA content of heart muscle samples normalised to GAPDH mRNA. G. Relative obscurin protein expressed in whole homogenates of heart muscle. A) Western blots of whole heart muscle homogenates separated by SDS- PAGE (See Fig 2). Top Memcode protein stain, bottom Ob59 anti-obscurin antibody. B) Calculated relative obscurin levels using the method as shown in Fig 3. Results from individual lanes are shown. C) Relative proportions of the three obscurin bands in atrial and ventricular muscle homogenates. (PDF) [file pone.0138568.s001.pdf]

SUPPLEMENTAL MATERIAL

OBSCURIN *(OBSCN)* MUTATIONS ARE ASSOCIATED WITH FAMILIAL DILATED CARDIOMYOPATHY AND HAPLOINSUFFICIENCY

SUPPLEMENTAL MATERIAL AND METHODS

Human heart muscle

All patients and subjects were enrolled to studies with local ethical approval and all investigations conform to the principles of the Declaration of Helsinki.

We used tissue samples from explanted hearts in the Sydney Tissue bank. 30 patients with a diagnosis of familial DCM or idiopathic DCM requiring transplant at less than 25 years old were selected for sequencing and further studies (see supp A) . As controls we studied muscle from 6 donor hearts (see Supp B). Donor hearts had no history of cardiac disease and normal ECG and ventricular function and were obtained when no suitable transplant recipient was found. The functional characteristics of some of the donor heart samples have been previously reported.(1, 2).

In addition to the 1000 genomes database and Exon Variants Server (EVS), a control population was used to analyze the frequency of the variants identified. This population consists of a cohort of 460 patients previously treated with doxorubicin but which did not develop heart failure (3).

DNA extraction

25 mg of frozen tissue was equilibrated at room temperature, incubated with a Steel Ball (Qiagen 69989) in a tissue lyser at 30 Hz for 1 minute, lysed overnight using proteinase K and genomic DNA has been isolated according to the manufacturer's protocol (Qiagen; Dneasy Blood-Tissue extraction).

RNA extraction

Extractions were performed with 25 mg of heart tissue with the RNA easy kit from Qiagen as described by the manufacturer, except for the lysis step. Lysis has been performed using the tissuelyzer at 50Hz during 2 minutes for a complete lysis). After

lysis, RNA extraction has been performed using the Qiagen RNA easy blood and tissue kit according to standard protocol. Reverse transcription was performed using the SuperScript™ II Reverse Transcriptase from Invitrogen as described by the manufacturer with 100ng of input RNA.

q-RT-PCR

cDNA was diluted in water (1:10) and 4mL of diluted cDNA were used as template in each assay. The q-PCR reaction mix is composed of 1mL of reverse and forward primer at an initial concentration of 2.5mM each and 12.5mL of SyberGreen (Invitrogen) and 6.5 mL of RNAse-free water.

The thermal cycle is composed of three steps 10 minute of denaturation at 94C, 35 cycles with a denaturation step of 30 sec at 94, an annealing at 55 for 45 sec, an elongation at 72 for 30 sec. The third step is a melting curve step to ensure the specificity of the reaction. All assays have been done in triplicate.

The analysis was performed with the relative 2^{DCt} method assuming that the reaction is efficient and that the quantity is doubled at each cycle and that GAPDH is the calibrator.

The following were used : Obscurin -F :ACTGGTGTGGTTAAGGGAGG,
Obscurin- R: AGAGCTGCCAGACATTCAGT,
GAPDH- F:AATCCCATCACCATCTTCCA,
GAPDH-R :TGGACTCCACGACGTACTCA

Whole genome sequencing

For whole-exome sequencing, we fragmented 1 µg of DNA with sonification technology (Bioruptor, diagenode, Liège, Belgium). The fragments were end-repaired and adaptor-ligated, including incorporation of sample index barcodes. After size selection, we subjected the library to an enrichment process with the SeqCap EZ Human Exome Library version 2.0 kit (Roche NimbleGen, Madison, WI, USA). The final libraries were sequenced on an Illumina HiSeq 2000 sequencing instrument (Illumina, San Diego, CA, USA) with a paired-end 2×100 bp protocol.

On average, 7 Gb of sequence were produced, resulting in 30-times coverage of more than 80% of target sequences (44Mb). We filtered primary data according to signal purity with the Illumina Realtime Analysis software.
We analysed the whole exome data for potential disease causing variants in 58 genes implicated in inherited cardiomyopathies, shown in Supp C using the filters indicated.

1000genomes database and Exon Variants Server, EVS

http://browser.1000genomes.org/Homo_sapiens/Info/Index has been used to analyse the frequency of the variants identified.

The current EVS data release (ESP6500SI-V2) http://evs.gs.washington.edu/EVS/ is taken from 6503 samples (a set of 2203 African-Americans and 4300 European-Americans unrelated individuals, totaling 6503 samples i.e 13,006 alleles). The samples included in the ESP6500 were selected from the populations of different studies and include controls, the extremes of specific traits (LDL and blood pressure), and specific diseases (early onset myocardial infarction and early onset stroke), and lung diseases. The goal of the ESP dataset is to release the frequency counts of specific variants without regard to phenotype.

The fact that patients with heart and lung diseases are the main source of the EVS data base does not allow the exclusion of a disease causing role of a variant if this variant is present in the data base.

Pyrosequencing

We confirmed the predicted mutations in D4, D14, D20 and D21 and tested for their presence in our cohort of 460 samples (3)(see Table 1). SNP genotyping by pyrosequencing was performed on an automatic PSQTM PSQ HS96A pyrosequencing instrument (Qiagen, Hilden, Germany). Pyrosequencing assays for SNP genotyping were designed using PSQ Assay Design software (Qiagen, Hilden, Germany). Primer sequences and assay conditions for the noncommercial pyrosequencing assays are available on request.

Pyrosequencing was performed according to manufacturer's instructions on a PSQ™ 96 HS instrument (Qiagen, Hilden, Germany) and analyzed by pyrosequencing software.

Gel electrophoresis

Whole tissue extracts were obtained for gel electrophoresis using T-Per Protein extraction reagent (Pierce, Thermo Scientific) including 1μg/ml E64, chymostatin and leupeptin protease inhibitors according to the manufacturer's protocol. The myofibrillar fraction of heart muscle was prepared by our standard protocol (1). Proteins were separated by two SDS-PAGE systems. High molecular weight proteins were separated on 2%agarose/2% polyacrylamide gels as described (4, 5). Other proteins were separated on 4-15% Polyacrylamide gradient gels (Criterion, Bio-Rad). Western blots were made in a wet blotting apparatus for 5 hours at 250 milliamps in the cold room. Blotted proteins were reversibly stained with MemCode Reversible stain kit (Thermo Scientific) and then probed with antibodies to Obscurin (OB13, Ob58 and Ob59, a gift from M.Gautel, King's College London (6)) and visualised with ECL pus (GE Healthcare).

Immunohistochemistry

Frozen sections of human heart tissue were cut using a Leica CM 1950 cryostat and were retrieved on poly-L-lysine coated slides. After drying they were fixed for 5 min in acetone at -20°C, washed in PBS and blocked with 5% preimmune goat serum in 1%BSA/GB (GB: 20 mM Tris, 155 mM NaCl, 2 mM EGTA, 2 mM MgCl2, pH7.5) for 30 min at RT. Incubations with primary and secondary antibodies were carried out in a humid chamber at RT, intermittent washing was done in PBS. As primary antibodies polyclonal rabbit anti obscurin antibodies (see above) were used in combination with either monoclonal mouse anti sarcomeric alpha-actinin (clone EA-53, Sigma) or monoclonal mouse anti myomesin (clone B4, Grove et al., 1984 PMID: 6537951). As secondary antibodies multilabelling quality Cy2-goat anti rabbit immunglobulins and Cy3-goat anti mouse immunoglobulins (from Jackson Immunochemicals via Stratech Scientific) were used in combination with DAPI (Sigma) to visualize the nuclei. Samples were mounted in 0.1M Tris/glycerol (3:7), 50 mg/ml N-propyl gallate pH9.5. Imaging was carried out using a 63x/1.4NA lens on a Leica SP5 confocal microscope equipped with a blue diode and argon and helium neon lasers.

LEGENDS to SUPPLEMENTAL FIGURES

A Table of muscle samples studied and putative disease-causing mutations found.

B Patient data for the donor heart muscle used as controls in this study.

C WES sequencing protocols: left , list of genes tested for mutations, right filter conditions used to find the potential disease-causing mutations

D Alignment of Obscurin B isoforms in human, mouse, mole rat and turtle. Only the regions of the sequence with mutations are shown

E Domain diagram of the two large muscle isoforms of obscurin. Location of mutations and the epitopes of Ob13, Ob58 and Ob59 are shown

F Relative Obscurin mRNA content of heart muscle samples normalised to GAPDH mRNA

G Relative obscurin protein expressed in whole homogenates of heart muscle

A) Western blots of whole heart muscle homogenates separated by SDS-PAGE (See Figure 1). Top Memcode protein stain, bottom Ob59 anti-obscurin antibody.

B) Calculated relative obscurin levels using the method as shown in Figure 2. Results from individual lanes are shown.

C) Relative proportions of the three obscurin bands in atrial and ventricular muscle homogenates.

NHLI ID	Sydney ID	Gene	Mutation	Diagnosis	Sex	Age	A or V	reference
D1	-	TNNC1	pG159D	FDCM	M	3	V	Kaski 2007(7)
D2	2.008	TNNI3	pK36Q	FDCM	M	15	V	Carballo 2009(8)
D3	2.059	None found		FDCM	M	43	V	
D4	2.066	OBSCN<br>DSP	pE963K<br>pR1537C	FDCM	M	43	V	Xu 2010(9)
D5	2.081	None found		PP/FDCM	F	34	V	
D6	4.100	TTN	pR23464T fs*41	FDCM	M	22	V	
D7	4.125	TTN	pR23464T fs*41	FDCM	M	37	V	
D8	3.005	Sample not sequenced		FDCM<br>IHD	M	62	V	
D9	2.029	TTN	pY18923*	FDCM	F	22	V	
D10	5.054	None found		FDCM	F	43	V	
D11	4.121	None found		FDCM	F	29	V	
D12	4.047	MYOM1	pE247K	FDCM	F	63	V	
D13	1.093	TTN	p1118I fs*21	IDCM	M	37	V	
D14	3.070	OBSCN	pV2161D,<br>pF2809V	FDCM	M	17	V	
D15	3.111	MYH7	pE1426K	FDCM	M	43	V	Villard 2005(10)
D16	7.036	None found		IDCM	M	56	A	
D17	1.018	DSP	pR1392Q	IDCM	M	25	A	
D18	1.019	MYH7	pS1836Q	IDCM	M	25	A	
D19	1.028	VCL	pA413T	IDCM	M	24	A	Lopes 2013(11)
D20	1.041	OBSCN	pD5966N	FDCM	M	56	A	
D21	1.059	OBSCN<br>SCN5A	pR4856H<br>pS216L	FDCM	M	43	A	Marangoni 2011(12)
D22	1.097	TCAP	pR106C	FDCM	M	19	A	Hirtle-Lewis 2013(13)
D23	2.007	TTN	pW14974*	IDCM	M	22	A	
D24	2.041	None found		FDCM<br>IHD	M	56	A	
D25	2.059	None found		FDCM	M	43	A	
D26	2.115	MYH7<br>GLA	pE1610Q<br>D313Y	FDCM	F	54	A	Froissart 2003(14)
D27	3.096	PLB	p39*	IDCM	F	23	A	Haghighi 2003(15)
D28	3.133	TTN	pN16499K fs*5	FDCM	F	60	A	
D29	4.066	PKP2	pI531S	FDCM<br>IHD	M	52	A	denHaan 2009(16)
D30	5.066	LAMA4	pV1808I	PP/FDCM	F	34	A	

Titin amino acid numbering is based on UniProt Q8WZ42-11 Isoform, Obscurin numbering is based on Q5VST9

Supplement A

Biopsy sample	NH	NK	NM	NQ	NR	NS
Diagnosis	SAH	SAH	Hypoxic brain injury	ICH	Hypoxic brain injury	SAH
Age	48	45	23	37	18	55
Gender	F	M	M	M	M	F

Clinical details of the donor heart samples used in this study

SAH=subarachnoid haemorrhage, ICH= intracerebral haemorrhage

Myectomy sample	MD	MN	MV
Diagnosis	HCM	HCM	HCM
Age	48	49	53
Gender	M	M	M
mutation	None found	None found	MYBPC3 IVS17+5G>T

Clinical details of the myectomy samples used in this study

HCM = hypertrophic cardiomyopathy

Obscurin supp B

ABCC9	GATAD1	PLN
ACTA1	GLA	PRKAG2
ACTC1	JUP	RBM20
ACTN2	LAMA4	RYR2
ANKRD1	LAMP2	SCN5A
BAG3	LDB3	SGCD
CASQ2	LMNA	TAZ
CAV3	MYBPC3	TCAP
CRYAB	MYH6	TMEM43
CSRP3	MYH7	TMPO
CTF1	MYL2	TNNC1
DES	MYL3	TNNI3
DMD	MYLK2	TNNT2
DSC2	MYOM1	TPM1
DSG2	MYOM2	TTN
DSP	MYOZ2	TTR
DTNA	NEBL	VCL
EMD	NEXN	ZBTB17
EYA4	OBSCN	
FHL2	PKP2	

Genes analysed for FDCM mutations

Filter Parameter	Values
GrpA: allele frequency range	25-100
GrpA: variations per gene	1-10
GrpA: gene burden fraction	filter off
GrpA: deNovo probability	filter off
GrpA: overlapps ROH	NO
GrpB: allele frequency range	25-100
GrpB: variations per gene	1-10
GrpB: gene burden fraction	filter off
GrpB: overlapps ROH	NO
Minimal read coverage	6
Minimal variation quality	10
GATK best practice min VQSLOD	-8
IonTorrent Haloplex Panel	filter off
Exclude Mpileup only INDELs	filter on
Maximal number seen in Epilepsy InhouseDB (n=511)	10
Minimal variation frequency	1%
Maximal number seen in Structural InhouseDB (n=611)	10
cnMops copy number states	0,1,3
ExomeDepth copy number states	0,4
Maximal target distance	100
Transcript biotypes	protein_coding
Variation locations	COMPOSED;GENES;INTRON;CDS
Transcript types	CCDS_Refseq
Variation types	DEL;SNP;INDEL;INS
Only include HGMD professional	filter off
Min RVIS percentile	filter off
Consequence types	Protein structure affected;Strong 5'SS/3'SS effects
Regions	
dbSNP	
Genes	ABCC9;ACTA1;ACTC1;ACTN2;ANKRD1;BAG3;CASQ2;CAV3;CRYAB;CSRP3;CTF1;DES;DMD;DSC2;DSG2;DSP;DTNA;EMD;EYA4;FHL2;GATAD1;GLA;JUP;LAMA4;LAMP2;LDB3;LMNA;MYBPC3;MYH6;MYH7;MYL2;MYL3;MYLK2;MYOZ2;NEBL;NEXN;OBSCN;PKP2;PLN;PRKAG2;RBM20;RYR2;SCN5A;SGCD;TAZ;TCAP;TMEM43;TMPO;TNNC1;TNNI3;TNNT2;TPM1;TTN;TTR;VCL;ZBTB17
Exclude Genes	

Filter parameters for identifying obscurin gene variants

WES sequencing data: left , list of genes tested for mutations, right filter conditions used to find the potential disease-causing mutations

Obscurin supp C

```
Human      SP|Q5VST9|OBSCN_HUMAN  SSSKVCMEATGCTRRLVVQQAGQADAGEYSCEAGGQRLSFHLDVK--------------- 976
Mouse      SP|A2AAJ9|OBSCN_MOUSE  SSSKVRMEASGCSRRLVVQQAGKADAGEYSCEAGGQKLSFHLDVT--------------- 1317
mole rat   TR|G5C996|G5C996_HETGA ASSRVRVEATGCRRQLVVQQAGEADAGEYSCEAGGRKISFRLDVTGCVAVAEWAETWRPC 992
turtle     TR|M7C5P7|M7C5P7_CHEMY SSRKFKVESEGKSRRLVVEQVEKKDAGEYICEAAGQKLTFKITVT--------------- 1199
                                  :* :. :*: *  *:***:*. : ***** ***.*::::*:: *.

           SP|Q5VST9|OBSCN_HUMAN  MHTLTLSGLRPEDSGLMVFKAEGVHTSARLVVTELPVSFSRPLQDVVTTEKEKVTLECEL 2189
           SP|A2AAJ9|OBSCN_MOUSE  VHILTLSALQPQDSGLVAFRAEGVHTSARLIVTELPVSFTRVLQDVVATQKEKVTLECEL 2622
           TR|G5C996|G5C996_HETGA VHSLTLSGLRPQDSGLVAFRAEGVQTSARLVVTELPVTFSRPLQDVVVTQKEKVTLKCEL 2280
           TR|M7C5P7|M7C5P7_CHEMY KHSLTLSSLKLEDSGLISFKAEGIHSSGRLIVTELPVKISKALVDIKAPEKDKVTFECEL 2503
                                   * ****.*: :****: *:***:::*.**:******.::: * *: . :*:***::***

           SP|Q5VST9|OBSCN_HUMAN  RKYILVVREAAPSDAGEVVFSVRGLTSKASLIVRERPAAIIKPLEDQWVAPGEDVELRCE 2849
           SP|A2AAJ9|OBSCN_MOUSE  RKYTLTVKDAADSPAGEVVFSVLGLTSKASLIIRERPVDITKPLEDQRTTLGEDVMLSCE 3281
           TR|G5C996|G5C996_HETGA RKYMLTVRDAAVGDTGEVVFSVRGLTSKASLLVRERPVAIVKPLEDQQAEPGEDVVLSCE 3000
           TR|M7C5P7|M7C5P7_CHEMY RKYTLDIRNVVAADAGEVVFTARNLNSKASLVVKEKPAEIIKQLENKTAAAGQDISLSCE 3216
                                  *** * :::.. .*:******:.  *.*****:::*:*. * * **:: .  *:*: * **

           SP|Q5VST9|OBSCN_HUMAN  TYLKKAGRPGTSPLASKVGAPAAPSVKPQQQQEPLAAVRPPLGDLSTKDLGDPSMDKAAV 4877
           SP|A2AAJ9|OBSCN_MOUSE  TYLKKAGGPGISPLASKHEAQVTTSVKPQKQQEPVVPTCPPPGDLSAADLMDPSLDKAAV 5817
           TR|G5C996|G5C996_HETGA TYLKKAGRPGASLLARQLGAPAAPSVKSQRQQEQPATVHPPPGDSSAEGLGDPSMDRAAV 7808
           TR|M7C5P7|M7C5P7_CHEMY ------------------------------------------------------------

           SP|Q5VST9|OBSCN_HUMAN  LSSIDLNDQVEGDDRAFEVWQEREDSVRKYLLQARTAIIKSSWVKEICGIQQRLALPVWR 6012
           SP|A2AAJ9|OBSCN_MOUSE  LSSIDLNDQVEGDDRAFEVWHEREDSVRKYLLQARTVIIKNSWVKEICGIQQRLAQPVWR 6952
           TR|G5C996|G5C996_HETGA LSSIDLNDQVEGDDRAFEVWHEREDSVRKYVLQARTTVIKSSWVKEICGIQQRLALPMWS 8939
           TR|M7C5P7|M7C5P7_CHEMY LSHIDVNDLVEGDDRAFEIWHEREDSVRKYLLQARTVIIKNSWVKEICGIQQRISLPVWI 6856
                                  ** **:** *********:*:*********:*****.:**.************:: *:*
```

D4 D14 D14 D21 D20

Alignment of sequences for OBSCN gene from human, mouse, mole rat and turtle (human ObscurinB, UniProt Q5VST9-1,is 7968 amino acids. RefSeq NM-001098623)

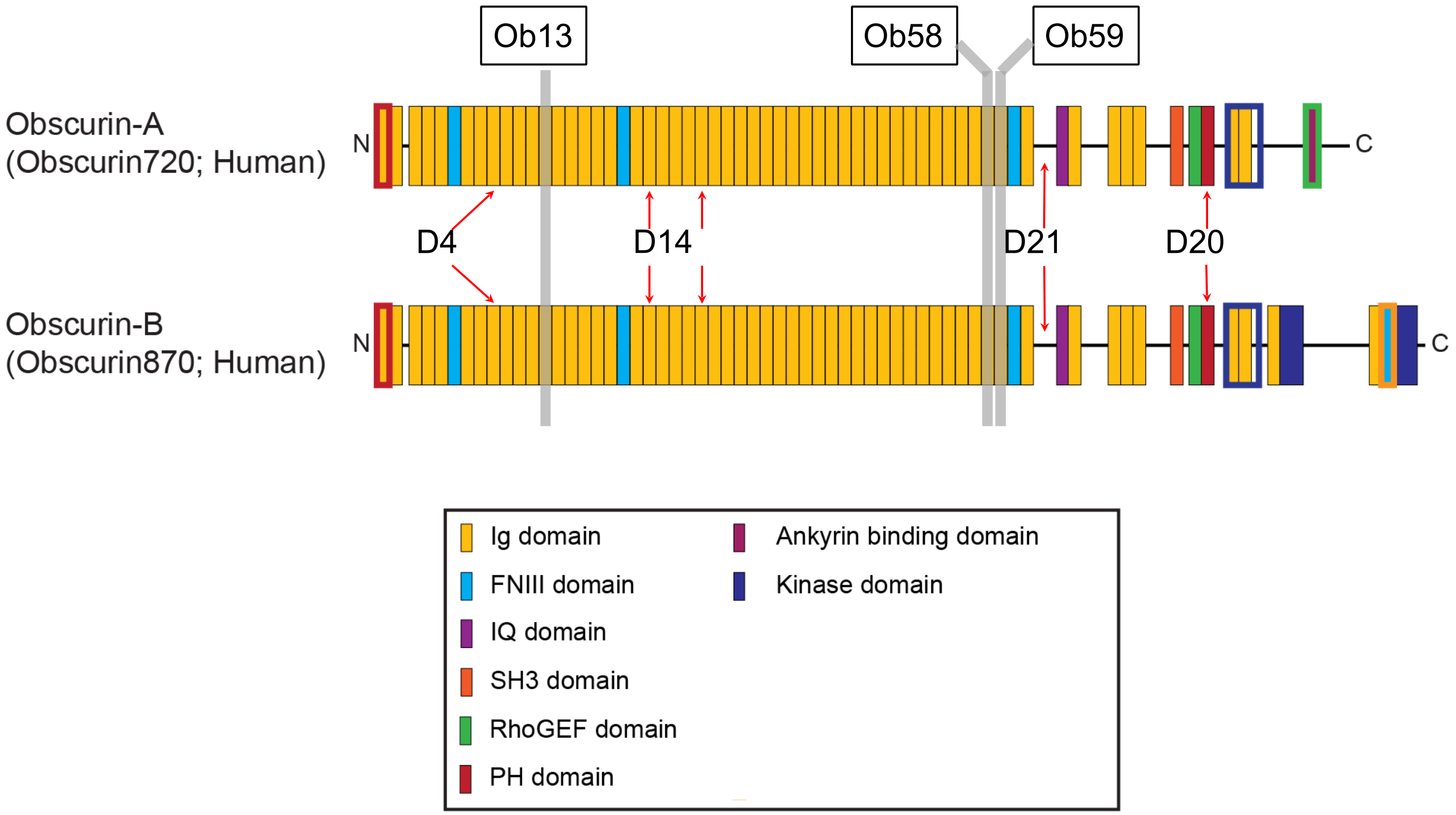

Location of mutations and antibody epitopes in the domain structure of the long obscurin isoforms

figure adapted from Ackerman et al (2014) DOI: 10.1371/journal.pone.0088162

Obscurin Supp E

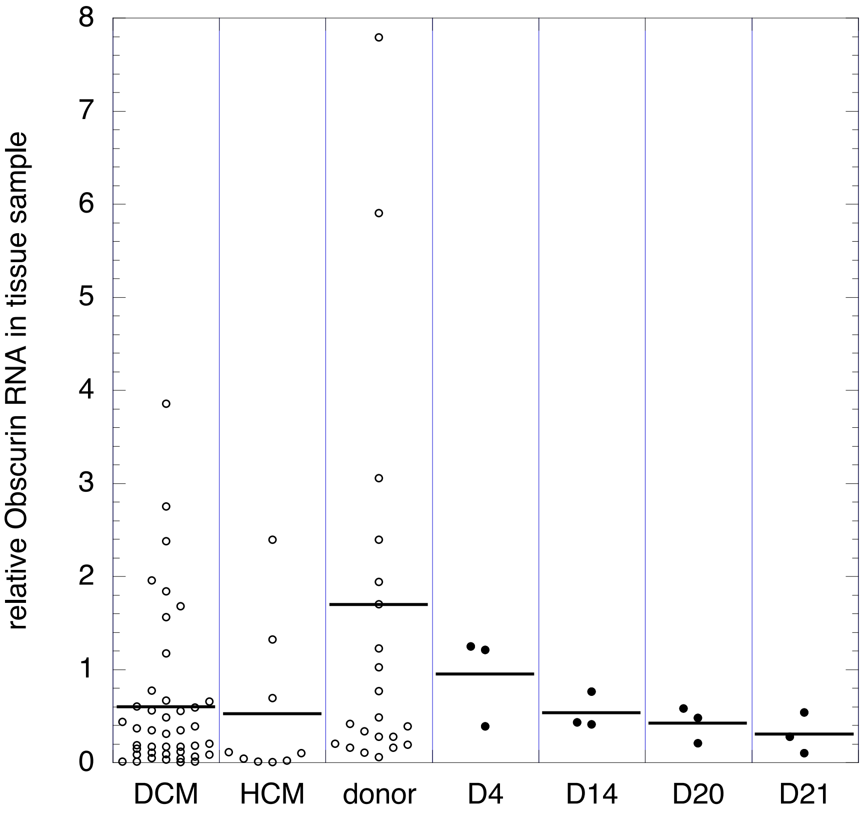

Relative Obscurin mRNA content of heart muscle samples normalised to GAPDH mRNA

Obscurin supp F

A

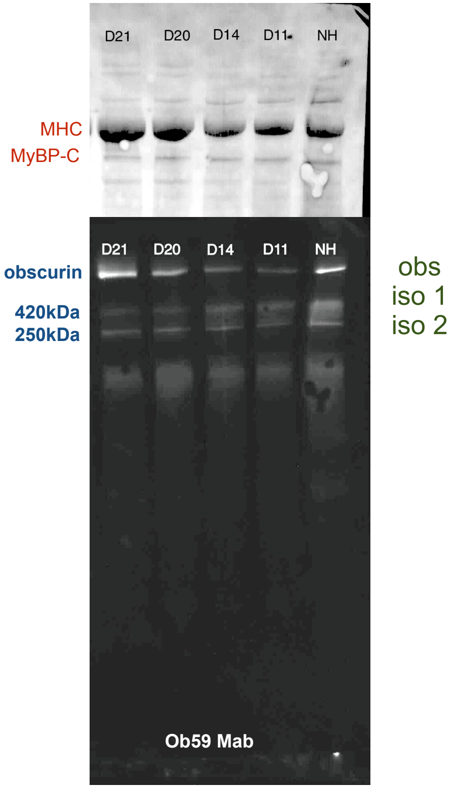

B

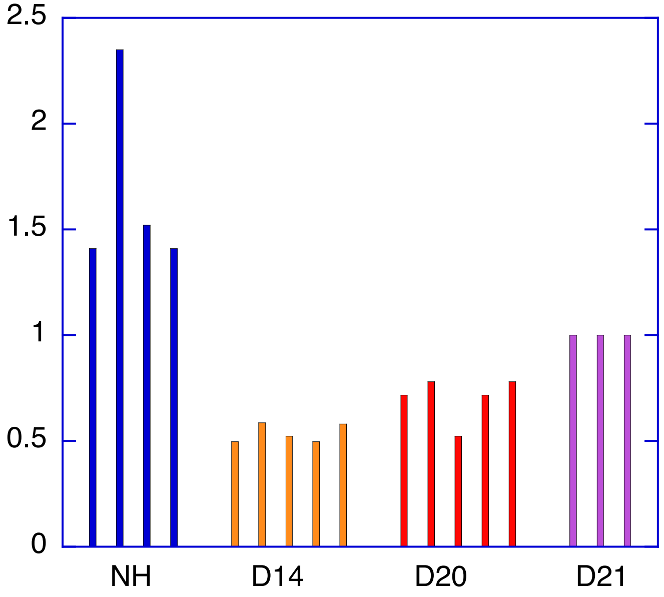

Quantitity of obscurin protein relative to α-actinin, normalised to D21. Calculated from 5 gels similar to the one shown here

C

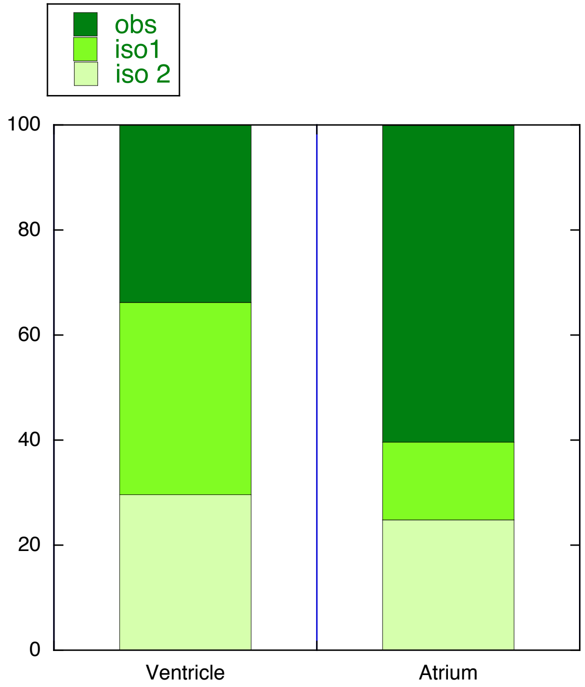

Relative quantities of the three isoforms of obscurin detected in whole human heart muscle homogenates of atrium and ventricle

Obscurin supp G

REFERENCES

1 Messer, A.E., Jacques, A.M. and Marston, S.B. (2007) Troponin phosphorylation and regulatory function in human heart muscle: Dephosphorylation of Ser23/24 on troponin I could account for the contractile defect in end-stage heart failure. *J Mol Cell Cardiol*, **42**, 247-259.

2 Bayliss, C.R., Jacques, A.M., Leung, M.-C., Ward, D.G., Redwood, C.S., Gallon, C.E., Copeland, O., Mckenna, W.J., Dos Remedios, C., Marston, S.B. *et al.* (2012) Myofibrillar Ca2+-Sensitivity Is Uncoupled From Troponin I Phosphorylation In Hypertrophic Obstructive Cardiomyopathy Due To Abnormal Troponin T. *Cardiovasc Res*, **97**, 500-508.

3 Knoell, R., Postel, R., Wang, J., Kraetzner, R., Hennecke, G., Vacaru, A.M., Vakeel, P., Schubert, C., Murthy, K., Rana, B.K. *et al.* (2007) Laminin-alpha4 and integrin-linked kinase mutations cause human cardiomyopathy via simultaneous defects in cardiomyocytes and endothelial cells. *Circulation*, **116**, 515-525.

4 Tatsumi, R. and Hattori, A. (1995) Detection of giant myofibrillar proteins connectin and nebulin by electrophoresis in 2% polyacrylamide slab gels strengthened with agarose. *Anal Biochem*, **224**, 28-31.

5 Warren, C.M., Krzesinski, P.R. and Greaser, M.L. (2003) Vertical agarose gel electrophoresis and electroblotting of high-molecular-weight proteins. *Electrophoresis*, **24**, 1695-1702.

6 Young, P., Ehler, E. and Gautel, M. (2001) Obscurin, a giant sarcomeric Rho guanine nucleotide exchange factor protein involved in sarcomere assembly. *J Cell Biol*, **154**, 123-136.

7 Kaski, J.P., Burch, M. and Elliott, P.M. (2007) Mutations in the cardiac Troponin C gene are a cause of idiopathic dilated cardiomyopathy in childhood. *Cardiol Young*, **17**, 675-677.

8 Carballo, S., Robinson, P., Otway, R., Fatkin, D., Jongbloed, J.D., de Jonge, N., Blair, E., van Tintelen, J.P., Redwood, C. and Watkins, H. (2009) Identification and functional characterization of cardiac troponin I as a novel disease gene in autosomal dominant dilated cardiomyopathy. *Circ Res*, **105**, 375-382.

9 Xu, T., Yang, Z., Vatta, M., Rampazzo, A., Beffagna, G., Pilichou, K., Pillichou, K., Scherer, S.E., Saffitz, J., Kravitz, J. *et al.* (2010) Compound and digenic heterozygosity contributes to arrhythmogenic right ventricular cardiomyopathy. *J Am Coll Cardiol*, **55**, 587-597.

10 Villard, E., Duboscq-Bidot, L., Charron, P., Benaiche, A., Conraads, V., Sylvius, N. and Komajda, M. (2005) Mutation screening in dilated cardiomyopathy: prominent role of the beta myosin heavy chain gene. *European Heart Journal*, **26**, 794-803.

11 Lopes, L.R., Zekavati, A., Syrris, P., Hubank, M., Giambartolomei, C., Dalageorgou, C., Jenkins, S., McKenna, W., Consortium, U.k., Plagnol, V. *et al.*

(2013) Genetic complexity in hypertrophic cardiomyopathy revealed by high-throughput sequencing. *J Med Genet*, **50**, 228-239.
12 Marangoni, S., Di Resta, C., Rocchetti, M., Barile, L., Rizzetto, R., Summa, A., Severi, S., Sommariva, E., Pappone, C., Ferrari, M. *et al.* (2011) A Brugada syndrome mutation (p.S216L) and its modulation by p.H558R polymorphism: standard and dynamic characterization. *Cardiovasc Res*, **91**, 606-616.
13 Hirtle-Lewis, M., Desbiens, K., Ruel, I., Rudzicz, N., Genest, J., Engert, J.C. and Giannetti, N. (2013) The Genetics of Dilated Cardiomyopathy: A Prioritized Candidate Gene Study of LMNA, TNNT2, TCAP, and PLN. *Clin Cardiol*, **36**, 628-633.
14 Froissart, R., Guffon, N., Vanier, M.T., Desnick, R.J. and Maire, I. (2003) Fabry disease: D313Y is an alpha-galactosidase A sequence variant that causes pseudodeficient activity in plasma. *Mol Genet Metab*, **80**, 307-314.
15 Haghighi, K., Kolokathis, F., Pater, L., Lynch, R.A., Asahi, M., Gramolini, A.O., Fan, G.C., Tsiapras, D., Hahn, H.S., Adamopoulos, S. *et al.* (2003) Human phospholamban null results in lethal dilated cardiomyopathy revealing a critical difference between mouse and human. *J Clin Invest*, **111**, 869-876.
16 den Haan, A.D., Tan, B.Y., Zikusoka, M.N., Llado, L.I., Jain, R., Daly, A., Tichnell, C., James, C., Amat-Alarcon, N., Abraham, T. *et al.* (2009) Comprehensive Desmosome Mutation Analysis in North Americans With Arrhythmogenic Right Ventricular Dysplasia/Cardiomyopathy. *Circulation: Cardiovascular Genetics*, **2**, 428-435.
